# Morphological diversity of *Quercus* fossil pollen in the northern South China Sea during the last glacial maximum and its paleoclimatic implication

**DOI:** 10.1371/journal.pone.0205246

**Published:** 2018-10-12

**Authors:** Lu Dai, Qinghe Hao, Limi Mao

**Affiliations:** 1 Department of Geography and Spatial Information Techniques, Ningbo University, Ningbo, China; 2 State Key Laboratory of Palaeobiology and Stratigraphy, Nanjing Institute of Geology and Palaeontology, CAS, Nanjing, China; 3 State Key Laboratory of Marine Geology, Tongji University, Shanghai, China; 4 CAS Key Laboratory of Economic Stratigraphy and Palaeogeography, Nanjing Institute of Geology and Palaeontology, Chinese Academy of Science, Nanjing, China; Union College, UNITED STATES

## Abstract

We aimed to obtain high resolution vegetation data and climate information about the LGM in the inland of the northern SCS based on key pollen types. Dominant *Quercus* fossil pollen grains in the core from the continental shelf of the northern SCS have been identified at the infrageneric level by using scanning electron microscopy. Based on tectum ornamentation, we recognized five sculpture types of *Quercus* pollen, namely, rodlike, rodlike masked, rodlike vertical, verrucate and micro-verrucate. Such a high diversity of *Quercus* fossil pollen types indicated that broadleaved forests were widely distributed in the inland along the northern SCS and included species of the subgenera *Cyclobalanopsis* and *Quercus*, of which subgenus *Cyclobalanopsis* populations were highly dominant. Low abundance of deciduous *Quercus* pollen probably derived from temperate-subtropical forests, while abundant evergreen pollen types of subgenura *Quercus* and *Cyclobalanopsis*, as well as other pollen of broadleaved taxa in the pollen assemblages, strongly suggest that the inland has been covered by dense subtropical forests. Consequently, the warm and humid subtropical climate prevailed during the LGM in the inland along the northern SCS. Our results shed new light on regional climatic conditions during the LGM in eastern Asia based on high diversity of *Quercus* fossil pollen in marine deposits from northern SCS.

## Introduction

Various climate interpretations based on pollen data have been proposed in previous studies for the inland of the northern SCS during the LGM, from temperate climate [1–3] to middle subtropical and southern subtropical climate [4–8]. Such debatable interpretations may be attributed to the lack of reliable pollen proxy data (e.g., indicator of temperature). In some cases, pollen signals of tropical and subtropical forests were masked by low pollen identification rate, low representation of arboreal pollen, and overrepresentation of nonarboreal pollen in tropical setting [9]. For instance, nonarboreal pollen was extremely rich during the LGM [1–2, 10]. In contrast, herbs such as Poaceae and Cyperaceae are not convincible climate indicators due to their broad ecological habitats and the fact that herb habitats are normally controlled by local environmental conditions in tropical and subtropical China [11]. Fortunately, some arboreal pollen (e.g., *Quercus* sensu lato) can be a good indicator of temperature and precipitation owing to improved pollen identification, particularly in monsoon regions in eastern Asia. Therefore, detailed understanding of past forest components and their dynamics based on detailed pollen identification may refine the paleoecological and paleoclimatic reconstruction at regional scale.

In the northern SCS, *Quercus* pollen is a dominant arboreal component in the sediments from the LGM, with the percentages of ~20% in the northern continental shelf and Chaoshan Plain [6, 12] and 20%–40% in the Leizhou Peninsula [7, 13–14]. *Quercus* pollen (mainly of the subgenus *Cyclobalanopsis*) is a good indicator of the south-subtropical and tropical climate in China [11]. Pollen morphological investigations in both extant species and fossil types confirmed that *Quercus* pollen can be divided into evergreen and deciduous types [15], which are then further subdivided into several groups [16–20]. Therefore, infrageneric taxonomy of *Quercus* pollen can provide useful references for accurate reconstruction of past forest communities and associated regional climate in the northern SCS.

Some studies have highlighted the significance of infrageneric identification of *Quercus* fossil pollen by using SEM. For instance, identification of *Quercus* subgenus *Lepidobalanus* pollen at the species level revealed the changes in forest composition since the LGM [21]. In the Himalayan area, the fossils of *Q*. *semecarpifolia* and *Q*. *incana* were distinguished in the pollen diagram, indicating the shifts between cold and warm climate [22]. Morphological and ultrastructural observations of *Quercus* fossil pollen in the Sea of Japan were highly accurate in recognizing oak pollen at the species level, in which *Quercus* pollen was considered a crucial proxy for tracing vegetation changes during the Holocene [23].

**Fig 1:**
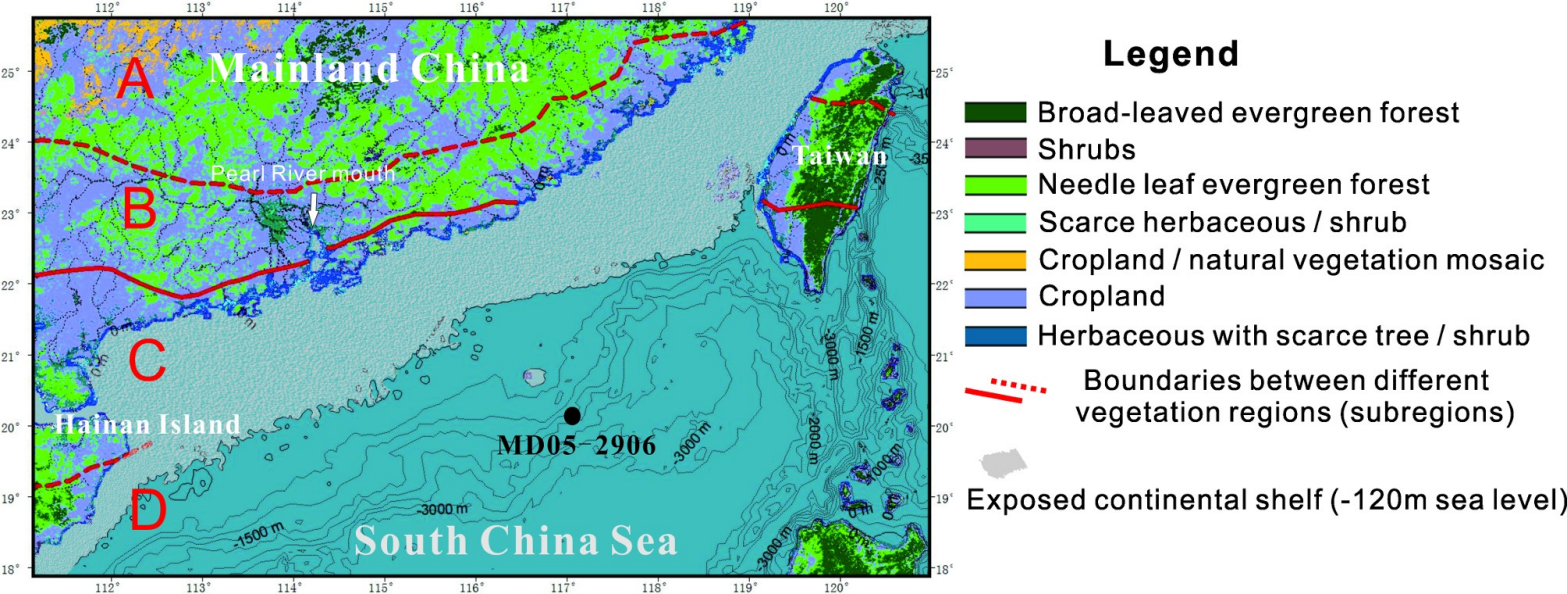
Modern vegetation map with vegetation belts. (A) Middle subtropical evergreen broadleaved forest, (B) southern subtropical evergreen broadleaved forest, (C) northern tropical semi-evergreen forest and tropical moist forest, and (D) southern tropical monsoon forest and tropical moist rainforest. Maximum exposed continental shelf during the period of low sea level (-120 m) is represented by the white shaded area. The figure is modified from [6]. The digital vegetation map was downloaded from http://bioval.jrc.ec.europa.eu, and the vegetation belts are based on [26].

China is home for more than 110 species of the subgenus *Quercus* and 77 species of the subgenus *Cyclobalanopsis* (Flora of China, www.eflora.cn). Many plant physiologists suggested that *Quercus* sensu lato include the subgenera *Quercus* and *Cyclobalanopsis* [24–25]. Subgenus *Quercus* includes evergreen and deciduous life forms, with the former distributed in the tropical and subtropical regions, and the latter mainly distributed in the temperate regions. Subgenus *Cyclobalanopsis* is widely distributed in subtropical and tropical regions of China [26] (Fig 1).

In this study, we focused on *Quercus* fossil pollen grains extracted from the LGM sediments from the MD05-2906 core located in the northern SCS [6]. Pollen of subtropical and tropical taxa, primarily composed of *Quercus* pollen, is a dominant arboreal component of the pollen record of the MD05-2906 core. The present study implemented SEM to improve pollen identification accuracy and yield new regional climatic information during the LGM in eastern Asia based on the diversity of *Quercus* fossil pollen from northern SCS.

## Material and methods

The northern SCS is bordered by southern mainland China, Hainan Island, and Taiwan Island, and it is connected to the East China Sea and Pacific Ocean by the Taiwan and Bashi Straits (Fig 1). The regional vegetation is generally characterized by tropical and subtropical broadleaved evergreen forests composed mainly of Fagaceae, Lauraceae, Theaceae, Hamamelidaceae, and Magnoliaceae [26]. The tropical vegetation elements gradually increase as the latitude decreases. The zonal vegetation changes from middle subtropical evergreen broadleaved forest to south tropical monsoon forest and tropical moist rainforest in southeastern China (Fig 1).

*Quercus* fossil pollen grains were extracted from the sediment core MD05-2906 (20°08.16′N, 117°21.59′E) that was drilled in the continental slope near Dongsha Island at a water depth of 1,636 m (Fig 1). On the basis of previously published pollen assemblages, for SEM analysis we chose *Quercus* fossil pollen found at a depth of 15.03–15.11 m and dated at ~20,200–18,800 cal BP (LGM period) [6]. *Quercus* fossil pollen was from two sediment samples, sample thickness was 2 cm.

*Quercus* pollen samples were treated with hydrochloride and hydrofluoric acid. Forty-one pollen grains were analyzed; No more new types of pollen sculpture can be found when the last several pollen grains were identified. All *Quercus* pollen grains were of tricolpate or tricolporate type. For SEM observation, pollen grains were transferred onto aluminum stubs with adhesive carbon tape and coated with gold. A Hitachi S-4800 cold field emission SEM was run at 8 kV and 7 μA and with working distances of about 9 mm. All observations were carried out at the Ningbo Institute of Industrial Technology, Chinese Academy of Sciences. Most of *Quercus* pollen samples shown in the figures are labeled with the original test numbers in the laboratory. Pollen terminology and group classification of *Quercus* pollen were based on references [17, 27].

We confirm that the field studies did not involve endangered or protected species.

## Results

Infrageneric groups of *Quercus* pollen, as well as their type, evergreen or deciduous, were tried to determined based on the comparisons with size and sculpture of modern oak pollen.

### Overall morphological comparison of *Quercus* fossil pollen

**Fig 2:**
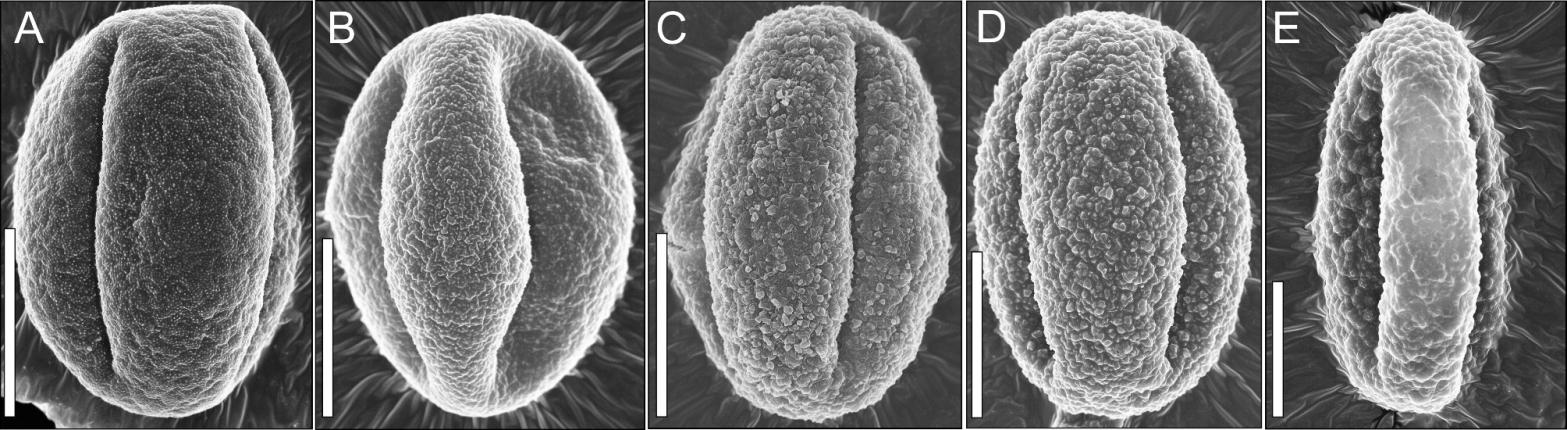
Comparison between typical exine sculptures of oak fossil pollen in MD05-2906 core. Scale bar = 10 μm.

The length of pollen polar axis ranged from 20 μm to 25 μm, the equatorial diameter ranged from 15μm to 20 μm (Fig 2A–D); one pollen is very large, the length of polar axis is about 30 μm (Fig 2E). Under magnification ×4500–5000, we observed pollen with different surfaces, and found sculptures various from fine to very coarse (Fig 2A–2E).

### Fine structures of the tectum in *Quercus* fossil pollen

#### Rodlike structure

**Fig 3:**
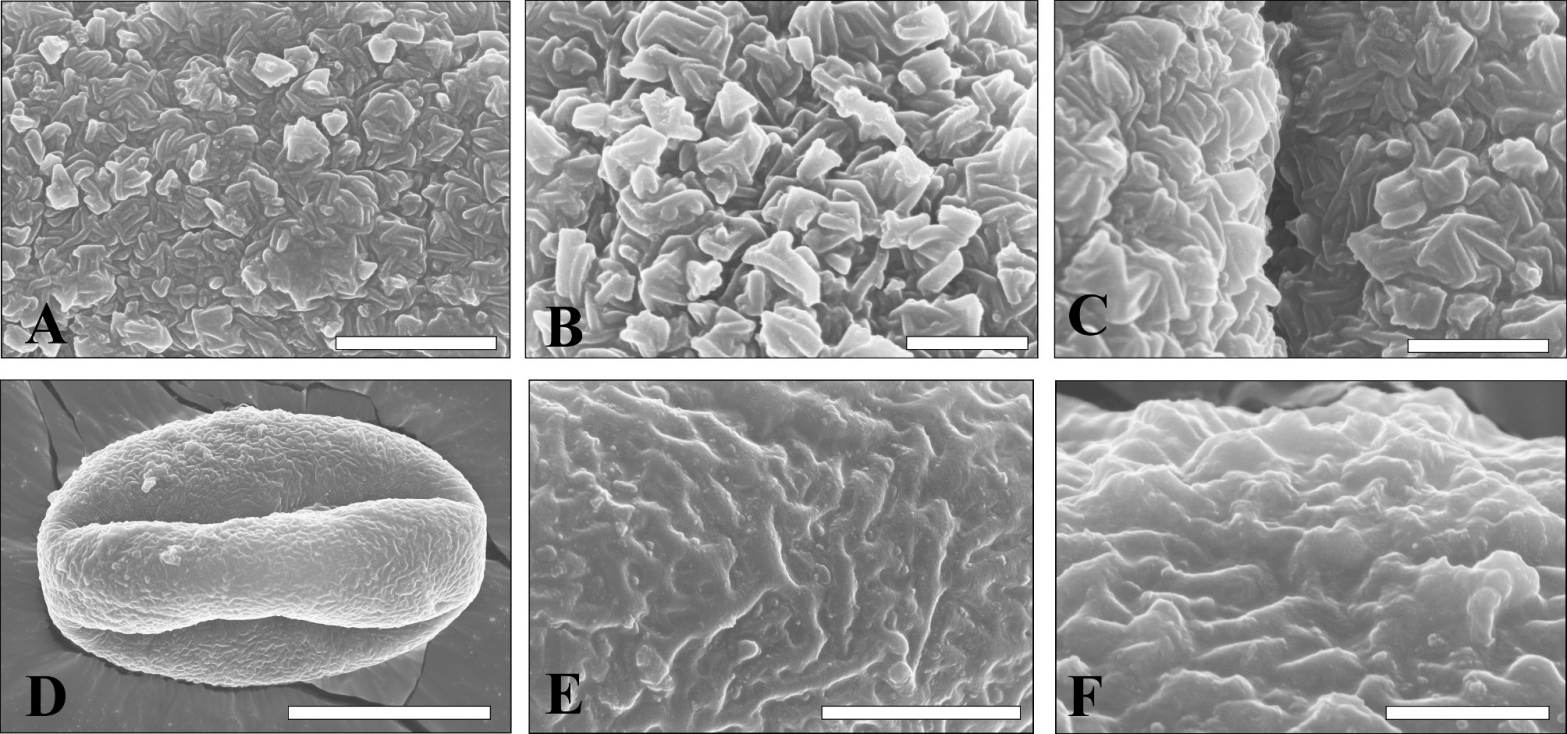
Rodlike ornamentations. A-C are from No. 0089–03 sample that is also shown in Fig 2C; D-F are from No. 0089–13 sample. Scale bar = 10 μm in D, 2 μm in A and E, 1 μm in B, C and F.

Two pollen grains were found in this sculpture type, which were possibly derived from different species: 1) the first one is a typical rod-shaped structure. This type of pollen was characterized by agglomerated structure formed of rodlike elements oriented in different directions (Fig 3A–3C). The agglomerations are about 0.25 μm high and 0.3–0.5 μm wide. This structure may be the basic unit of tectum ornamentation of *Quercus* pollen extine [17, 28]. 2) the second one has rugulate structure composed of strip-like elements, the surface is psilate (Fig 3D–3F). The sculpture may have resulted from the fused rodlike elements, because the rod-shaped structure consisting of elongated sexine elements more than 1 μm long is distinct. Rodlike structure is typical in the *Quercus* infrageneric group *Ilex* [17].

#### Verrucate and micro-verrucate structure

**Fig 4:**
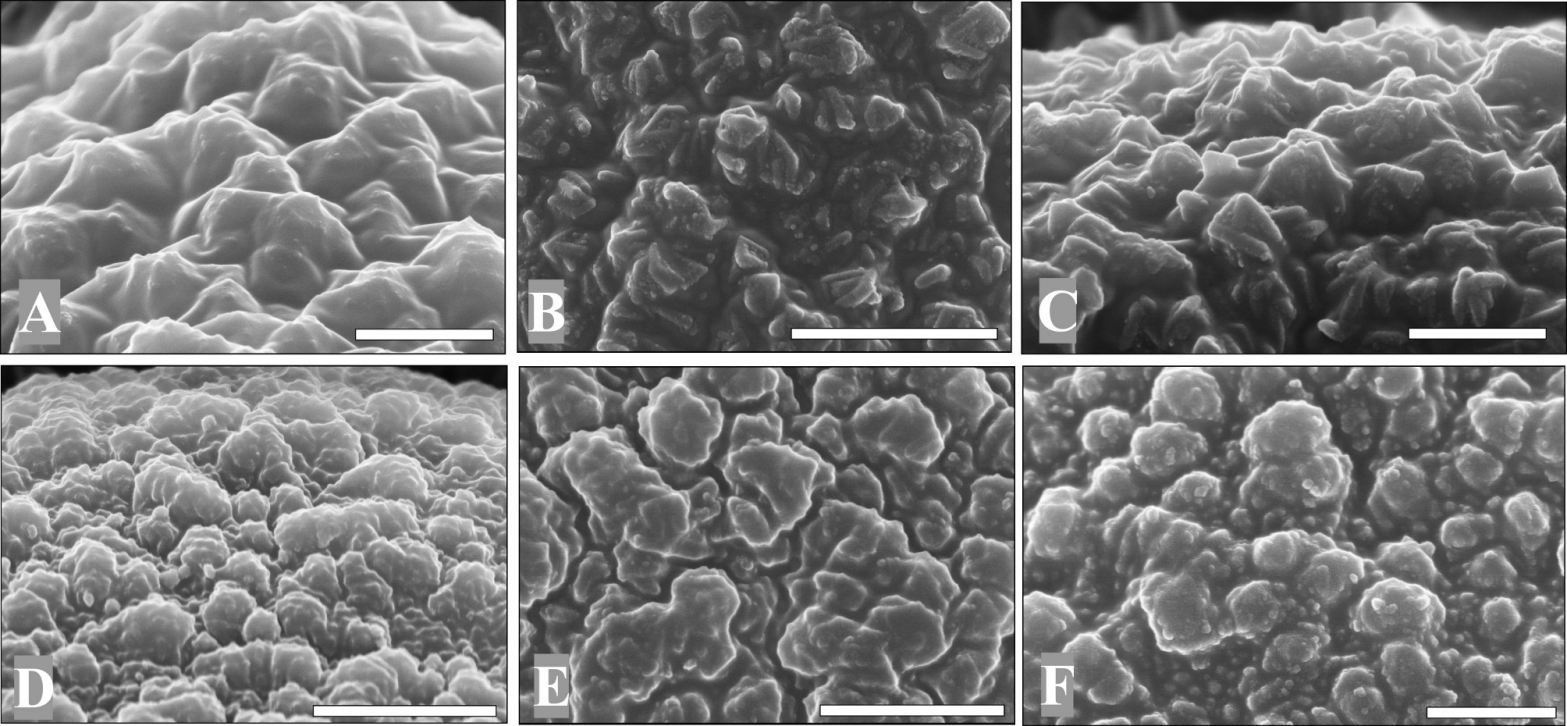
Verrucate and micro-verrucate ornamentation. A is from No. 0104–03 sample (corresponding to Fig 2E), B and C are from No. 0155–07 sample (corresponding to Fig 2D). D-E are from No. 0123–01 sample (corresponding to Fig 2B). F is from No. 0155–04 sample. Scale bar = 2 μm in B and D, 1 μm in A, C, E and F.

Fourteen pollen grains were found in both sculpture types. Verrucate sculpture is formed by masking of rod-shaped structures by secondary accumulation of sporopollenin in the tectum; the rod-shaped elements are faint and scattered agglomerations are more distinct than those in the rodlike structure (Fig 4A–4C and S1 Fig). This sculpture is very similar to the *Quercus* infrageneric group *Cerris* [17]. In the type of verrucate pattern, two species were possibly identified: 1) one pollen is with very scabrate verrucate sculpture, consisting of numerous large agglomerations; the base diameter of agglomerations is 0.1–1μm, the length of pollen polar axis is about 30 μm which is larger than that in other pollen (20–25μm) (Figs 2E and 4A). 2) two pollen grains have a relatively small verrucate sculpture, where the base diameters of agglomerations are 0.1–0.6 μm and rod-shaped elements are more distinct. The length of polar axis are 20–25 μm (Figs 2D, 4B and 4C).

As sporopollenin further accumulates in the tectum of pollen with verrucate structure, rod-shaped elements become largely fused and invisible, forming a micro-verrucate structure (Fig 4D–4F and S2 Fig). Tuft agglomerations fuse to form islands that are elongated or rounded, forming a cauliflower-like structure (Fig 4D–4F). This sculpture corresponds to the *Quercus* infrageneric group *Quercus* [17].

#### Rodlike vertical structure

**Fig 5:**
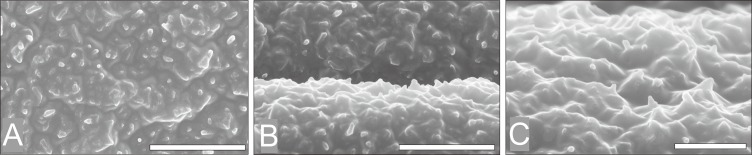
Rodlike vertical ornamentations. Scale bar = 2 μm in A and B, 1 μm in C.

Six pollen grains were found in this type. These pollen grains have a rod-shaped vertical sculpture in which the rod-shaped structures are masked by accumulated sporopollenin in the tectum and only the upper parts of the tufts are visible (Fig 5A–5C). The axis of tufts is more perpendicular to the pollen surface and needle-like protrusions are present that are about 1/3 μm in length. This sculpture corresponds to the *Quercus* infrageneric group *Cyclobalanopsis* [17].

#### Rodlike masked structure

**Fig 6:**
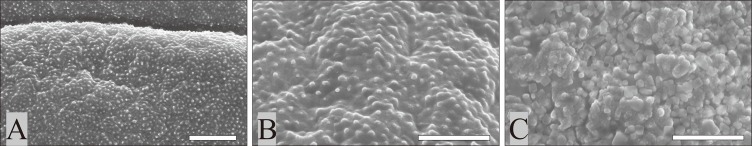
Rodlike masked ornamentations. A and B are from No. 0155–05 sample (corresponding to Fig 2A). C is from No. 0166–3 sample. Scale bar = 2 μm in A, 1 μm in B and C.

Nineteen pollen grains were found in this type. Rod-shaped structures are largely masked by accumulated sporopollenin so that the most apical parts of the tufts are visible on the tectum surface (Fig 6A and 6B).

In this group, one pollen found is different from others. Its tectum is composed of numerous small particles, pollen surface is relatively psilate (Fig 6C).

## Discussion

### Oak species diversity during the LGM in the northern SCS

In this study, we have identified five types of *Quercus* pollen and possible eight species in the marine sediments. Their morphology was compared with that of modern *Quercus* pollen to determine to the species level. Current studies have shown that ornamentation of *Quercus* pollen can be used for accurate identification up to the level of infrageneric groups or sections but not species [16–17, 19–20, 29]. Therefore, we focused on distinguishing the subgenera *Quercus* and *Cyclobalanopsis*, as well as evergreen and deciduous types of pollen, as these elements will provide valuable insight into the reconstruction of forest communities in the northern SCS during the LGM.

Previous research has shown that *Quercus* infrageneric group *Ilex* with rodlike sculpture is evergreen; group *Ilex* includes *Q*. *acrodonta*, *Q*. *aquifolioides*, *Q*. *baronii*, *Q*. *cocciferoides*, *Q*. *dolicholepis*, *Q*. *franchetii*, *Q*. *engleriana*, *Q*. *gilliana*, *Q*. *guyavaefolia*, *Q*. *kingiana*, *Q*. *lanata*, *Q*. *longispica*, *Q*. *monimotricha*, *Q*. *pannosa*, *Q*. *rehderiana*, *Q*. *semecarpifolia*, *Q*. *senescens*, and *Q*. *spinosa* distributed in south and southwest China [17, 19]. The fact that these species are all evergreen suggests that the group *Ilex* is indicative of the evergreen subgenus *Quercus* (Fig 3) [15].

Pollen with verrucate ornamentation is attributed to the *Quercus* infrageneric group *Cerris* [17]. In this group, most of species are deciduous, such as *Q*. *acutissima* and *Q*. *variabilis* (pollen sizes are 25.2–37.8 × 21–37.8 μm and 26.2–37.8 × 25.2–33.6 μm), which is widely distributed in northern subtropical China [16]. A few species are semi-evergreen or evergreen, such as *Q*. *crenata* and *Q*. *suber*, which are absent in China and Southeast Asia [30]. Pollen with micro-verrucate structure was attributed to the *Quercus* infrageneric group *Quercus*, derived from the subgenus *Quercus* [17]. This group includes pollen from mostly deciduous species such as *Q*. *aliena*, *Q*. *dentata*, *and Q*. *fabrei*, which are distributed in temperate and subtropical China [15, 17].

Recently, a number of morphological analyses of *Quercus* pollen have been carried out in the close region. In the Sea of Japan, *Quercus* fossil pollen with verrucate and micro verrucate ornamentation was attributed to deciduous type [18]. In eastern China, scabrate-verrucate sculpture was described as a major diagnostic character of deciduous oak pollen; this sculpture was also occurred in some evergreen pollen types, however, most of which were distributed in the Mediterranean region [15].

In our samples, most of pollen with verrucate and micro-verrucate sculpture is relatively small in size (~25 μm) and likely not of a typical deciduous type. However, the diagnostic character of pollen sculpture strongly suggested that both pollen types belonged to deciduous.

The rodlike vertical structure is typical for the infrageneric group *Cyclobalanopsis* [20]. Many species in the subgenus *Cyclobalanopsis* have this type, such as *Q*. *lamellosa*, *Q*. *lobbii*, *Q*. *glauca*, *Q*. *schottkyana*, *Q*. *delavayi*, and *Q*. *gambleana* [20].

Pollen with a rodlike masked structure is attributed to the section *Protobalanus*, most of which is mainly distributed in the New World [17]. However, many species with such sculpture belong to the subgenus *Cyclobalanopsis*, such as *Q*. *augustinii*, *Q*. *glauca* var. *hypargyrea*, *Q*. *myrsinifolia*, *Q*. *sessilifolia*, and *Q*. *utilis* in China [20]. Therefore, we infer that the pollen with rodlike masked structure probably belongs to the subgenus *Cyclobalanopsis*.

**Fig 7:**
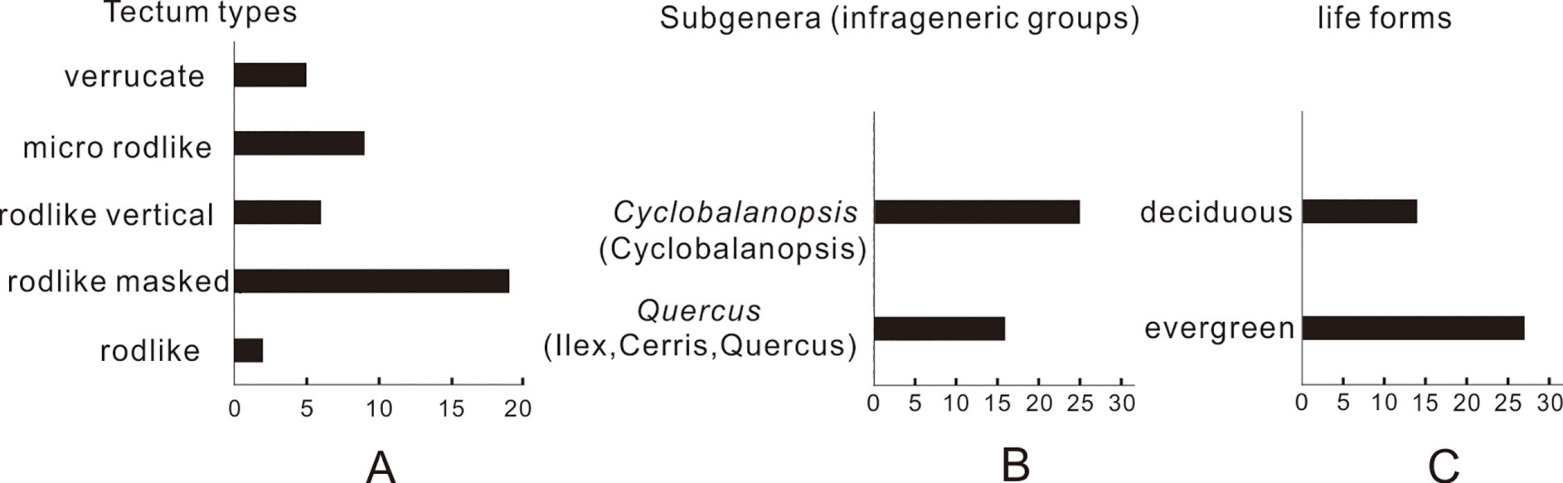
Numbers of *Quercus* pollen grains found in different tectum types, subgenus taxa, and life forms.

In China, grouping the *Quercus* fossil pollen into evergreen and deciduous types is significant for interpreting the Quaternary dynamics of temperate and subtropical climate [31]. Liu et al. summarized that evergreen oak pollen in subtropical China has rod-like, uniformly fine granules, scabrate-verrucate, and rugulate sculpture [15]. The first two types of sculpturing are mostly limited to evergreen oaks and can be used to identify fossil evergreen oak pollen, while the last two types are also found in many deciduous oaks. We confirmed similar results and found three types of sculpture patterns, namely rodlike, rodlike vertical, rodlike masked (Fig 7A). These pollen types belong to *Ilex* and *Cyclobalanopsis* groups, respectively (Fig 7B), corresponding to the evergreen oak. Meanwhile, pollen with verrucate and micro-verrucate sculptures possibly belongs to deciduous species (Fig 7A–7C).

### Forests and paleoclimatic reflection about the inland of northern SCS during the LGM

#### Forests in the inland of northern SCS during the LGM

This study revealed diverse oak species and associated forest communities. In the *Quercus* pollen assemblage, pollen of subgenus *Cyclobalanopsis* was dominant, accounting for 59% of total pollen (Fig 7A and 7B). Subgenus *Cyclobalanopsis* and subgenus *Quercus* pollen with rodlike and rodlike masked sculptures were evergreen (66%) (Fig 7C); therefore, such pollen types indicate the presence of subtropical forests.

Presently, Fagaceae is an important family in southern subtropical, tropical monsoon forest, and tropical rainforest of China. Among those, subgenus *Quercus* and subgenus *Cyclobalanopsis* are minor components in the northern SCS [24]. For instance, *Castanopsis*, Lauraceae, *Ficus*, *Schima*, *Syzygium*, Palmae, and ferns are dominant components in the Dinghushan National Nature Reserve (a typical broadleaved evergreen forest) located in the southernmost Mainland China [32]. Field investigation confirmed only the scattered presence of subgenus *Cyclobalanopsis* plants along a humid valley. Therefore, subgenera *Quercus* and *Cyclobalanopsis* plants are poor present in rainforests. A few subgenus *Cyclobalanopsis* communities composed of *C*. *fleuryi*, *C*. *championi*, and *C*. *bambusifolia* are narrowly distributed in the montane rainforest on Hainan Island in south China [33] (Fig 1).

Although subgenera *Quercus* and *Cyclobalanopsis* are not structural species of the forests, their pollen has a good representation, comprising 20%–40% of the pollen count in the montane rainforest on Hainan Island [34]. Subgenus *Quercus* (including subgenus *Cyclobalanopsis*) was a dominant broadleaved component (~20%) in the northern SCS; its pollen was derived from south Mainland China or the exposed continental shelf [8, 35]. Because structural species such as Lauraceae have low pollen representation, the high presence of *Quercus* pollen (mainly subgenera *Cyclobalanopsis* type) is a good indicator of subtropical forests [11].

In the glacial stratigraphy of the MD05-2906 core, the percentage of evergreen oak pollen attained 10%–20%; species richness was high and subgenus *Cyclobalanopsis* pollen type was dominant. Meanwhile, other tropical components, such as *Castanopsis*, Altingiaceae, and Euphorbiaceae are also abundant [6]. This pollen assemblage is similar to that in surface sediments around the northern SCS, indicating the presence of south-subtropical or tropical forests [35]. Moreover, the presence of possible deciduous pollen in the core implied that forest components were not completely similar with those found in present forests; therefore, the climate had slightly cooled down during the LGM (Fig 7C).

In several pollen records located in the northern SCS, non-arboreal pollen dominated during the LGM [1–2, 6, 8]; such pollen types were probably derived from the exposed continental shelf. However, the co-existence of rich subgenus *Cyclobalanopsis* (including subgenus *Quercus*) and non-arboreal plants is impossible, which is because the former indicates dense forests and the latter originates in grassland or steppe environment, a combination of environments that has no comparable analogue in the current vegetation cover. Therefore, we suggested that pollen in the marine sediments may be derived from the different pollen source area; most of the arboreal pollen was possibly transported from the inland forests by the old Pearl River rather than by the exposures of the continental shelf; abundant herbs, such as Poaceae, Cyperaceae possibly distributed in the coast and swamp, indicated special environmental conditions in the exposed continental shelf.

In summary, high richness of *Quercus* fossil pollen indicates various forest communities, with subtropical forests occupying the hill regions around the northern SCS during the LGM.

#### Paleoclimatic reflection about the inland of northern SCS during the LGM

Until now, many palynological studies sought to reveal paleoclimatic conditions during the last glaciation in the northern SCS using drilling cores ODP 1144, SO 17940, MD05-2904, MD05-2906, and STD 235 [1–3, 6, 8, 10] and the lacustrine pollen documents from the Leizhou Peninsula [7, 36], Chaoshan Plain [12], and Huguangyan Maar Lake [13] in mainland China and the island of Taiwan [4]. Based on those palynological records, different interpretations have been proposed. *Artemisia* pollen, which is a typical temperate component, was present in almost all marine pollen records during the LGM, suggesting a marked decrease in temperature [1–3, 8]. In contrast, forests were stable and their components were not significantly modified by climate changes, but deciduous species were poorly represented [7, 36]. According to the detailed forest reconstruction in the present study, we conclude that subtropical (even south-subtropical) climate still prevailed although temperature moderately decreased during the LGM in the northern SCS.

## Conclusions

Low pollen identification rate has limited detailed interpretation of the past vegetation dynamics and climate changes. In this study, the morphology of *Quercus* fossil pollen types has been detailed investigated using SEM to reveal various patterns of the tectum, which significantly improved our palynological interpretation on forest composition and contemporary climate scenario. Our results refine the paleoclimatic reconstruction in the northern SCS, and the following conclusions could be made:

1. In the fossil pollen assemblages of the core MD05-2906, five types of *Quercus* fossil pollen can be recognized; they are mostly evergreen, with the subgenus *Cyclobalanopsis* being the dominant pollen type.
2. During the LGM, the high species richness of the subgenera *Quercus* and *Cyclobalanopsis* indicates the past presence of various forest communities in the northern SCS that are similar to modern subtropical forests.
3. The presence of some identified pollen types and possibly deciduous *Quercus* pollen indicates a moderate decrease in temperature. However, the climate might not have been largely modified and subtropical climate still prevailed during the LGM.
4. During the LGM, inland forests were composed of complex tropical and subtropical broadleaved trees, including rich evergreen oak species in the northern SCS. Meanwhile, a number of non-arboreal pollen on the exposed continental shelf was probably not the indication of temperate climate.

## Supporting information

S1 Fig Verrucate ornamentation. Scale bar = 10 μm in 1a-3a, 1 μm in 1b-3b.
(TIF)

S2 Fig Micro-verrucate ornamentation. Scale bar = 10 μm in 1a-11a, 1 μm in 1b-11b.
(TIF)

## Abbreviations

SCS: South China Sea
LGM: Last Glacial Maximum
SEM: Scanning Electron Microscope
